# Making stillbirths count, making numbers talk - Issues in data collection for stillbirths

**DOI:** 10.1186/1471-2393-9-58

**Published:** 2009-12-17

**Authors:** J Frederik Frøen, Sanne J Gordijn, Hany Abdel-Aleem, Per Bergsjø, Ana Betran, Charles W Duke, Vincent Fauveau, Vicki Flenady, Sven Gudmund Hinderaker, G Justus Hofmeyr, Abdul Hakeem Jokhio, Joy Lawn, Pisake Lumbiganon, Mario Merialdi, Robert Pattinson, Anuraj Shankar

**Affiliations:** 1 Department of Genes and Environment, Division of Epidemiology, Norwegian Institute of Public Health, P.O. Box 4404 Nydalen, N-0403 Oslo, Norway; 2 Department of Obstetrics and Gynaecology, University Medical Centre Groningen, University of Groningen, The Netherlands; 3 Department of Obstetrics and Gynaecology, University Hospital, Assiut, Egypt; 4 Department of Chronic Diseases, Division of Epidemiology, Norwegian Institute of Public Health, Oslo, Norway; 5 Department of Reproductive Health and Research, World Health Organization, Geneva, Switzerland; 6 National Center on Birth Defects and Developmental Disabilities, Centers for Disease Control and Prevention, Atlanta, GA, USA; 7 Reproductive Health Branch, United Nations Population Fund, Geneva, Switzerland; 8 Department of Obstetrics and Gynaecology, University of Queensland; 9 Mater Mothers' Research Centre, Mater Health Services, Brisbane, Australia; 10 Center of International Health, University of Bergen, Bergen, Norway; 11 Effective Care Research Unit, Eastern Cape Department of Health, Universities of the Witwatersrand and Fort Hare, South Africa; 12 Department of Community Health Sciences, Aga Khan University, Karachi, Pakistan; 13 Saving Newborn Lives, Cape Town, South Africa; 14 Department of Obstetrics and Gynecology, Faculty of Medicine and Public Health, Khon Kaen University, Khon Kaen, Thailand; 15 Department of Obstetrics and Gynaecology, University of Pretoria School of Medicine, Pretoria, South Africa; 16 Department of Nutrition, Harvard School of Public Health, Harvard University, Boston, USA

## Abstract

**Background:**

Stillbirths need to count. They constitute the majority of the world's perinatal deaths and yet, they are largely invisible. Simply counting stillbirths is only the first step in analysis and prevention. From a public health perspective, there is a need for information on timing and circumstances of death, associated conditions and underlying causes, and availability and quality of care. This information will guide efforts to prevent stillbirths and improve quality of care.

**Discussion:**

In this report, we assess how different definitions and limits in registration affect data capture, and we discuss the specific challenges of stillbirth registration, with emphasis on implementation. We identify what data need to be captured, we suggest a dataset to cover core needs in registration and analysis of the different categories of stillbirths with causes and quality indicators, and we illustrate the experience in stillbirth registration from different cultural settings. Finally, we point out gaps that need attention in the International Classification of Diseases and review the qualities of alternative systems that have been tested in low- and middle-income settings.

**Summary:**

Obtaining high-quality data will require consistent definitions for stillbirths, systematic population-based registration, better tools for surveys and verbal autopsies, capacity building and training in procedures to identify causes of death, locally adapted quality indicators, improved classification systems, and effective registration and reporting systems.

## Background

### Why count stillbirths?

The vast majority of stillbirths are preventable, and simple interventions could lead to healthy infants as a rich reward for the resources invested [1-6]. Mothers and families could be spared the emotional burden accompanying pregnancy loss, and societies could gain by reducing a major public health problem.

Being counted is essential. Stillbirths are estimated to account for more than half of the world's perinatal deaths, but only a fraction are registered in any health information system [7]. Stillbirths have been invisible in the World Health Organization (WHO) reports on the global burden of disease [8] and in the United Nations (UN) Millennium Development Goals and Targets. According to the most recent WHO reports on perinatal mortality, 90 countries worldwide lacked any kind of data on stillbirths [7,9]. Improvements in basic registrations of stillbirths are both possible and urgently needed [10]. The "Who Counts?" series in *The Lancet *argued forcefully for the need to build and strengthen civil registration and health information systems globally [11-14]. Systematic and reliable registration of stillbirths is crucial to any health care program planning in this field. Analysis and use of such data provide the fundamentals for accountability and funding. Prioritization of registration and analysis of neonatal deaths in the Millennium Development Goals led to a global effort resulting in significant gains in prevention [15]. Accurately counting stillbirths will similarly provide an opportunity to set specific goals, the first step toward any improvement.

**Figure 1:**
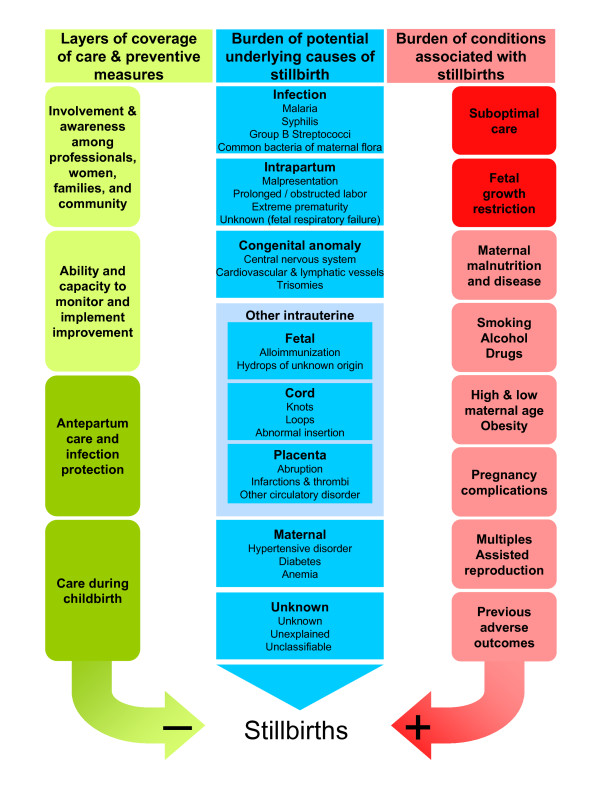
**Stillbirth determinants**. A framework of the setting and conditions that constitute the data sources needed for the understanding of stillbirth mortality. The classification of significant proportions of underlying causes of death globally is reproduced from CODAC simplified [50].

Simply being counted, however, is insufficient for planning, monitoring, and continuously improving efforts to prevent stillbirths. Additional resources and political commitment are needed to improve basic health care services and to overcome limited governance, infrastructure, and workforce. Localities with limited resources are also vulnerable to low-quality health services, where lasting and sustained improvement will likely come through a redesign of health care systems, not through continued funding of current failing systems. In such situations, accessible data are needed on core indicators of quality and availability of care, as well as on the prevalence of underlying causes of these deaths and associated conditions which constitute the framework for understanding stillbirths (Figure 1).

The need for better data is a pressing issue in stillbirth prevention, where there is a significant gap in translating knowledge into proven strategies to reduce fetal mortality [16-20]. Better data on stillbirths are also needed for basic research, which has been neglected. For every 67 publications on unexplained infant death (SIDS) in PubMed, there is only one on the far more prevalent problem of unexplained stillbirths (using the search terms ("unexplained stillbirth*" [All Fields] OR "unexplained fetal death*" [All Fields]) vs. ("Sudden Infant Death" [MeSH] OR "sudden infant death*" [All Fields] OR "SIDS" [All Fields])). This research gap is especially pronounced in low-income countries in which the burden of stillbirths is particularly high. The general lack of data and of research to understand and prevent stillbirths in low-income countries adds to the "10/90 gap"--the fact that less than 10% of research resources address conditions affecting more than 90% of the world's population [21]. Of all publications on stillbirth in PubMed, only about 3% relate to low-income countries (using the search terms (("stillbirth" [MeSH Terms] OR stillbirth* OR "fetal death" [MeSH Terms] OR fetal death*) AND humans [MeSH Terms])) with or without (AND ("developing countries" [MeSH Terms] OR developing countr*)).

Addressing the issue of counting stillbirths is clearly complementary to traditional approaches to perinatal data collection that have prioritized neonatal and maternal deaths. In a continuum of perinatal deaths, data on stillbirths emphasize the earlier phases of pregnancy and bring information that may convey benefits for both maternal and child health. Antepartum and intrapartum stillbirths are strong and direct indicators of quality of prenatal and obstetric care. Also, stillbirth rates, highly correlated to maternal death rates, continue to be a sensitive indicator in less affected communities where maternal deaths are too few to serve as a sensitive indicator [22].

Although counting stillbirths seems simple, it is complex in practice. The incentives for an individual to be counted in civil registration systems are linked to the perceived benefits [11]. For stillbirths, such incentives may not be obvious in communities where few advances in health care are offered. Apparent cultural resistance to registration can be considerable, making data collection problematic if context and culture are not sensitively addressed. In low-income countries, the conduct of even the most well-designed, population-based studies on stillbirths weighing more than 1000 g, has been met with difficulties as many stillborn infants are never weighed [10,18].

In this report, we assess how different definitions and limits in registration affect data capture, and we discuss the specific challenges of stillbirth registration, with emphasis on implementation. We identify what data need to be captured, we suggest a dataset to cover core needs in registration and analysis of the different categories of stillbirths with causes and quality indicators, and we illustrate the experience in stillbirth registration from different cultural settings. Finally, we point out gaps that need attention in the International Classification of Diseases (ICD) and review the qualities of alternative systems that have been tested in low- and middle-income settings.

## Discussion

### International definition and effects on data capture

**Figure 2:**
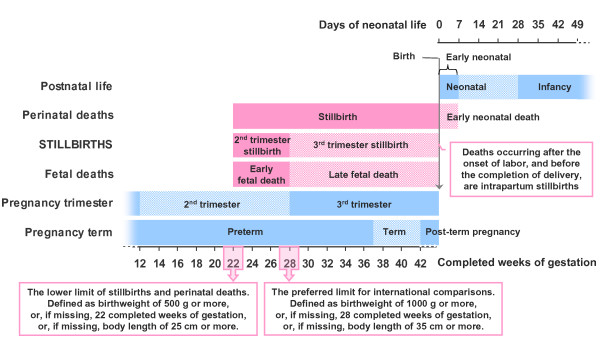
**Definition of stillbirth**. The definitions of stillbirth used by WHO and ICD in the framework of the continuum of perinatal losses and commonly used definitions of timing of pregnancy and newborn life. Categories of deaths in pink and periods of perinatal life in blue.

The WHO/ICD defines stillbirths as the death of a fetus that has reached a birth weight of 500 g, or if birth weight is unavailable, gestational age of 22 weeks or crown-to-heel length of 25 cm (Figure 2) [23]. The WHO also recommends using a higher limit (1000 g/28 weeks/35 cm) of third-trimester stillbirths for international comparisons [23]. Failure to adhere to WHO definitions and recommendations hampers stillbirth epidemiology. In the USA alone, reporting requirements are determined by individual states, and nine different definitions are used [24]; similar inconsistencies exist in Europe [25]. Reports that include higher rates of early stillbirths typically demonstrate higher rates of congenital anomalies, infections, and placental abruptions, which would suggest a need for different health care planning than in locations that report only third-trimester stillbirths [26-28].

Birth weight, thought to be more reliably reported, takes priority over gestational age in the WHO definition. Most high-resource countries have legislation related to stillbirth registration and reporting, with limitations almost uniformly based on gestational age ranging from 16 to 28 weeks [24,25,29]. In low-resource countries with scarce access to prenatal care and ultrasound dating of pregnancy, the accurate gestational age is seldom known. Differences in technical and cultural understanding of pregnancy duration adds to the problem, as both women and their care providers may count the number and variants of months (calendar, lunar or menstrual cycles) in various ways [30,31], traditional pregnancy calendars may not add up to 280 days (e.g. the traditional Chinese lunar calendar of ten pregnancy months represents 293 days), and an early spontaneous abortion may not be perceived as the end of a pregnancy but that gestation may be included in the duration of the subsequent pregnancy [32].

**Figure 3:**
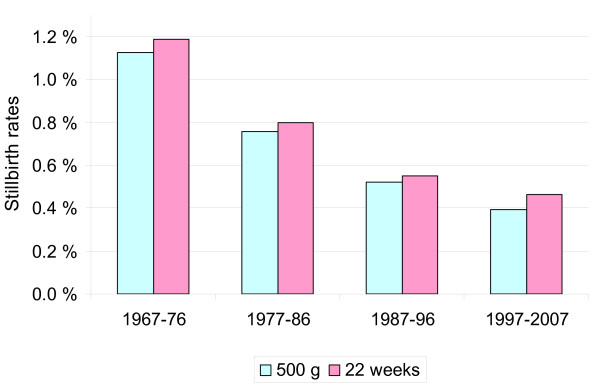
**Stillbirth rates by reporting criteria**. Norwegian stillbirth rates according to reports based on weight or gestational age. The data include 2.4 million births and 13,100 stillbirths in the Medical Birth Registry of the Norwegian Institute of Public Health.

Capture by birth weight yields lower stillbirth rates than capture by gestational age (Figure 3); therefore, a high-income country registering stillbirths after 22 weeks and subsequently reporting their numbers according to the 500-g birth weight limit will potentially underreport significantly. If these capture methods are applied to the Norwegian 1997-2007 data (compared with capture by either gestation or weight individually), 4.4% of stillbirths of ≥ 500 g occurring before 22 completed weeks of gestation are lost by the registration limits, and 18.6% of deaths after 22 completed weeks that weigh <500 g at birth are lost by reporting limits. In Australia, while reporting practices may also influence variations seen in reported stillbirth rates, nationally reported rates for 2006 from one agency (Australian Bureau of Statistics, http://www.ausstats.abs.gov.au) where birth weight (400 g) takes precedence over gestational age (20 weeks) are 30% lower than those from the other national agency (National Perinatal Statistics Unit, http://www.npsu.unsw.edu.au) where birth weight and/or gestation is used (5.2 v 7.4/1000, respectively). The true difference in stillbirth rates between high vs. low income countries is therefore larger than official statistics indicate. This gap will likely be greater in areas with higher rates of intrauterine fetal growth restriction, i.e., in low-resource regions. Underreporting will be an issue when registration limits are the same as the intended reporting limits. Hence, registrations should aim to document fetal mortality occurring earlier than the limits at which they aim to report for comparative purposes, e.g., 20 weeks/400 g and 26 weeks/750 g, to report accurately at 500 g and 1000 g, respectively [33].

### Identification of stillbirths

Beyond the heightened awareness of stillbirths needed to plan vital records or Demographic and Health Surveys to accommodate stillbirth registration in the many countries that lack any data on stillbirths, there are special issues that need to be addressed, providing both challenges and opportunities. An initial challenge is the ability of birth attendants in low-resource areas to identify an infant as stillborn. While the definition of fetal death requires that no heartbeat be present, there is often no ascertainment of such vital signs in settings where no resuscitation is attempted in the live-born but apparently lifeless newborn; such a newborn will be classified as stillborn if he or she does not recover spontaneously [34]. Higher coverage of the diagnostic tools and skills of neonatal resuscitation will improve correct registrations and, more importantly, prevent such apparent stillbirths [34,35].

Registration can only be successful if perceived as beneficial by the population being registered and by those responsible for collecting and reporting events. For the mother and her family, there may be no apparent benefits in terms of civil rights, health care, or other societal advantages normally awarded to mother and infant. In some settings, there are economic disadvantages for parents when a stillbirth is registered (Appendix 4). There is a lack of literature which directly assesses the factors that affect disclosure of stillbirths. However, areas that need attention to enable culturally sensitive data collection may be gleaned from practices in disclosure of pregnancy, the perception of stillbirth and the stillborn, and the context in which women make their health care choices.

In some cultures a new pregnancy is a celebrated event disclosed early in gestation, but a recurrent issue emanating from many low-resource settings is the risk associated with disclosure of pregnancy, which may outweigh perceived benefits. Not only is pregnancy in itself frequently seen as a state of shame and impurity [36], but the pregnant woman may see herself as a prime target for spirit-induced illness, witchcraft and other supernatural forces [37,38]. Such threats to herself and her baby may be perceived as a larger risk than biomedical causes [37], and since seeking health care may increase public exposure and risk, while biomedicine may not help against spirits [37-40], silence and hiding may be the first expressions of prenatal caring in their setting [37]. In the case of a pregnancy loss, disclosure may put both the woman's social status and future pregnancies at risk through community judgement of perceived "sins" which may include unfaithfulness, bewitching of her husband, possession by spirits or being a spirit wife of another man, illegal abortion or reproductive incapacity. Such community judgement may result in the women being abandoned or having to pay back the bride price [36,37,41]. Stillbirth is painful in all cultures, and the more problematic emotions are involved, and the less social support and acknowledgement of their loss received, the more complicated their grief [42-44]. Some parents affected by stillbirth receive massive support and recognition of their loss, but in different settings a woman's stillborn child may not be perceived as a significant "entity" and public displays of mourning may be culturally prohibited [36] - making any conversation about her stillbirth difficult. The need to assess the local perceptions of factors facilitating or obstructing stillbirth disclosure, and its ramifications, in planning the methods for data collection in a sensitive, private and confidential manner cannot be overestimated.

For the birth attendant, with first-hand knowledge of the event, there may be barriers to reporting as well. In most communities and for many reasons, health care professionals traditionally tend to underreport adverse events and outcomes, irrespective of whether they are objectively to blame for the event [45,46]. A blame culture with its inherent threat of punishment may result in the health professional's refuse to offer care to pregnancies with known complications, and reluctance to report adverse outcomes [40]. Despite a changing culture from individual blame to a system-approach to adverse events in many health care organizations [47], the health care professional attending births must still be expected to underreport intrapartum deaths unless provided with strong incentives. In communities where most births take place in institutions which provide maternity care, such barriers can potentially be overcome more easily, while they remain a major problem for stillbirths among women in rural areas who give birth at home. The objective lack of health care resources, accessibility and infrastructure, together with subjective maternal views of inadequate quality of care and lack of sensitivity to privacy and social, religious and cultural needs, will continue to prevent a large proportion of births from occurring in institutions in the foreseeable future.

Some of the challenges of capturing data on stillbirths in areas with high birth rates outside birthing institutions may be addressed by ensuring the registration of pregnancies before the perinatal period, if coupled with incentives such as access to antenatal care, allocation to maternity groups, dietary supplements, or other benefits - sufficient to outweigh the perceived risks of disclosing their pregnancy. This strategy will provide the most accurate denominator, unbiased by maternal or professional barriers, and also provide important opportunities for initial risk assessment and essential preventive measures. With sufficient incentives to register a live-born baby, special focus to document outcomes should be planned for those registered as pregnant but not presenting for birth registration. By registering the mother rather than the infant, pregnancy outcomes lost to follow-up may be captured at subsequent pregnancy registrations, as women experiencing loss have higher rates of renewed pregnancy than those having a live-born child. If health professionals clearly communicate that disclosure of previous stillbirths may aid in preventing recurrence, mothers may also find personal motivation to overcome obstacles in reporting their stillbirth.

### Causes and conditions to be captured

Considerable differences in causes and timing of stillbirths exist between populations, and these vary with the stillbirth rates. The setting and epidemiology of causes of death will define the most critical information on stillbirths needed to inform prevention and guide selection of the analyses that will provide the best indicators of improvement.

Longitudinal data from high-resource countries show that the rate of intrapartum stillbirths has fallen with improvements in intrapartum care [48]. Intrapartum death rates are therefore frequently used as an indicator of quality of care. As the rate of intrapartum stillbirths is directly associated with the availability of intrapartum care, a larger proportion of stillbirths occur in the intrapartum period in low- and middle-income than in high-income countries (31% vs. 16% of stillbirths, respectively) [48]. In Western Africa, the intrapartum stillbirth rate was estimated at 15/1000 in 2004 (36% of stillbirths), 50 times higher than the rate in North America of 0.3/1000 (10% of stillbirths) [7,49].

Preventability of intrapartum deaths will depend largely on gestational age, and timing of deaths should be registered both in terms of early versus late gestational deaths and, if known, whether the death occurred antepartum or intrapartum. Neither "intrapartum" nor "intrapartum asphyxia" are underlying causes of death but rather timing and final mechanism, respectively [50]. Such categories alone contain insufficient information to guide targeted improvements in care. When the most basic care is available, a majority of intrapartum deaths are caused by placental, cord, infectious, traumatic, and other specific complications, both in high- and low-resource countries [51-54]. Reports from Pakistan, Palestine, and South Africa all indicate that about one third of cases are linked to suboptimal care [53,55,56]. Characteristics and predisposing risks, such as plurality and maternal size, should be registered together with events and conditions presenting during the intrapartum period, as should information on the level and quality of care available and received, as indicated in the dataset template.

Irrespective of setting, the largest proportion of stillbirths are antepartum deaths. Although availability of a timely delivery is critically important to prevention, the pregnancy at risk for antepartum death, and the optimal timing of delivery to prevent it, must first be identified through antenatal care. Adverse pregnancy outcome can be most effectively anticipated, prevented, and treated through implementation of adequate antenatal care. Components of this care include counselling and initiation of health-promoting efforts, such as proper nutrition or insecticide-treated mosquito nets, combined with screening, identification, and monitoring of pregnancy risks [3-6].

The reported prevalence of major causes of antepartum deaths will differ between high- and low-resource communities, because of both their true prevalence and the coverage of adequate evaluation to identify and register the cause of death [41]. Adverse antepartum conditions are often complex and can be found in the mother, the fetus, the placenta, and the umbilical cord, and their interactions. On a global scale, syphilis, malaria, and other infections; congenital anomalies; placental abruptions and other placental insufficiencies; and pregnancy-induced hypertension cause most antepartum deaths [2,16,17,20,54,57-63]. Placental pathology, including those cases manifesting clinically as maternal hypertensive disorders, contribute to the underlying cause of death in 6 out of 10 stillbirths in low-income countries [49,64]. In low-resource settings, this condition may more often go untreated and progress to severe preeclampsia or eclampsia and be classified as such. Because of infrequent examinations of the placenta, such cases are likely to be classified as deaths of unknown cause associated with fetal growth restriction or maternal hypertensive disease rather than by the underlying placental cause. While an appreciation of the maternal and fetal conditions associated with placental pathology is important in risk reduction in both high- and low-resource country settings, lack of information about placental pathology still inhibits better understanding of etiology of these deaths.

The relative contribution of congenital anomalies to stillbirth rates in low-resource countries is low because of the high rates of other causes coupled with an under-diagnosis of malformations due to relying on findings from gross external malformations when autopsy and other diagnostic assessments are lacking. In countries with liberal abortion laws, however, the contribution of anomalies is often underestimated as a result of antenatal diagnoses and terminations of pregnancy before the perinatal period. In evaluating mortality rates associated with anomalies in communities with high coverage of prenatal ultrasound and access to medical termination of pregnancy, termination rates must therefore be taken into account [65].

The largest contributors to third-trimester stillbirths of infectious causes in the low-income world are malaria and syphilis [2,63], while typical pathogens in high-income countries are probably mostly missed in low-resource settings because of the specialized tests needed to identify them adequately, as in the case of group-B streptococci, Parvovirus B-19, *Listeria monocytogenes*, and early-gestation infections by common bacteria of maternal intestinal flora. Such undetected infections will also add to the group of antepartum stillbirths of unknown cause. Unknown cause of death may mean two different things in high- and low-income countries. There is a wide gap between unexplained stillbirths despite adequate post-mortem examinations, which represent one fourth of antepartum stillbirths in high-resource settings [66,67], and stillbirths of unknown cause resulting from a lack of information or examinations, which represent up to two thirds of antepartum deaths in communities where either suboptimal testing is performed or causes of death are poorly classified, both in high and low-income settings [68-70]. Addressing the challenges in evaluation of stillbirths of unknown cause, and prevention of unexplained stillbirths, requires that such entities are registered among causes, as discussed later.

Despite such differences, which make direct comparisons across populations difficult, low-resource communities should aspire to register their stillbirths in the same systematic manner as in settings with greater resources. A common classification system is needed to build a common understanding and bridge the knowledge gap. At the same time, it is essential for low-income countries to also collect basic data such as information on timing of death and fetal growth restriction in these same datasets.

### Datasets of stillbirths

**Figure 4:**
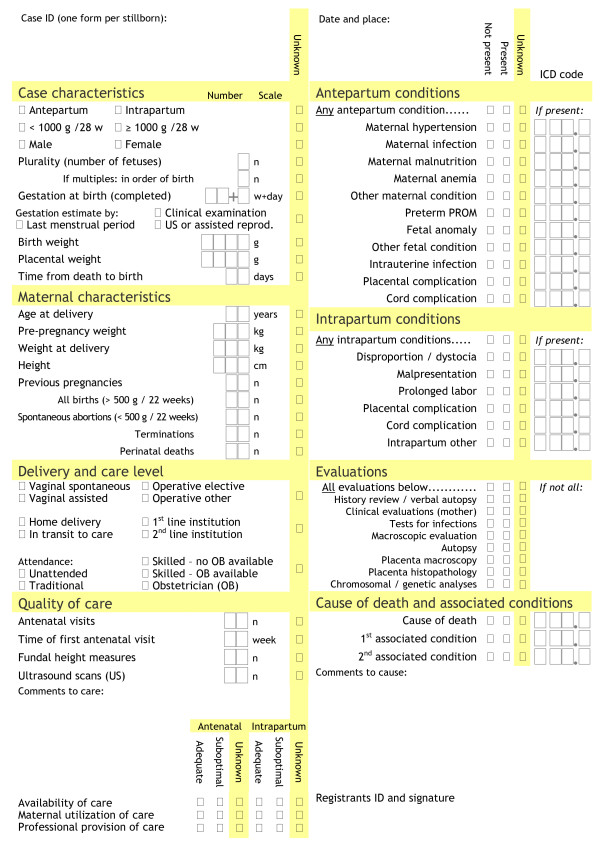
**Dataset template for stillbirths**. A template for the development of data collection forms for datasets of stillbirths.

Data on stillbirths should enable the analysis and monitoring of quality of care and the most prominent specific threats to reproductive health. Relying on "general improvement" of health and health care in the population will be insufficient. Reducing stillbirth rates in Scandinavia from levels currently seen in low-income countries to those seen today has taken more than 100 years [71]. Cause-specific interventions have been shown to be effective in reducing stillbirth rates [1,2,19,72]. The template dataset presented here (Figure 4) has been designed specifically to identify and monitor significant threats to pregnancy health that health care programs can address, both within quality of care and among the most prevalent causes of death and associated conditions globally. Examples of challenges and opportunities for such data collections are presented in Appendices 1, 2, 3 and 4.

In this template, the main underlying cause of death should be registered, and up to two associated conditions may be added to capture data on the scenario in cases with complex or multiple causes. It includes case details needed to analyze the potential for prevention and to correct for case mix when comparing populations. It also includes some of the basic measures of quality and availability of care, although more detailed information needs to be gathered to monitor specific interventions. In particular, measures of quality of care need to reflect local challenges to be helpful for further improvement [73-75]. National or local guidelines should define what is considered as adequate care versus suboptimal care, depending on which evidence-based interventions that can be provided [3-6], and this dataset segregates deficiencies according to whether suboptimal care was the result of lacking coverage (adequate care not available) or insufficient health care delivery and usage.

Most of the suggested data items are easily observed by a skilled birth attendant in most settings and will be sufficient for classification in main categories of causes of death, as illustrated in Figure 1. A large proportion of stillbirths do, however, occur in settings where the mother has received no antenatal care and gives birth at home with no skilled attendant present. In this situation, a verbal autopsy, taken after the birth, offers a potential source of helpful data. The abolition of pregnant women lacking any maternity health care is already targeted as the primary goal in any preventive strategy against maternal and perinatal deaths. Implementation of basic maternity health care to improve pregnancy outcome is not dependent on knowledge of specific causes of stillbirth within the setting within which it is delivered. Current knowledge is sufficient for remedial action, and the will and ability to change are the only things lacking to mobilize a call to action. Substandard situations must not scale down ambitions for better data collection; rather, the ability to identify causes of death should be scaled up. The template dataset provides the opportunity to register data items as unknown, and the frequency of unknown data should be monitored as a quality indicator together with the rate of stillbirths classified as cause unknown.

The current system for verbal autopsy does seem to provide useful information on the causes of death in most cases, but it still has unsolved issues for stillbirths [76,77]. With the most prevalent causes of death in stillbirths in mind, efforts to improve the value of verbal autopsy should be a priority. Ideally, some level of skilled post-mortem investigation, at least an external macroscopic examination, should be performed [78]. Birth attendants can be trained to use illustrations on posters to assess the degree of maceration and determine approximately when fetal death occurred, but international standards for verbal autopsy for unattended home deliveries resulting in stillbirth are lacking [79]. Tools to determine pathologies of the placenta for birth attendants in low-resource areas should be developed and tested. The tools would help birth attendants collect basic, but important, information such as placental-fetal birth weight ratio, gross description of placental dimensions and shape and umbilical cord insertion, color and smell indicating infections, detection of clots and sign of abruption, large infarcts, and widespread fibrosis. In settings where autopsies are never performed, protocols that include placental examination often provide valuable data for capturing the cause of death [61,80]. Regrettably, examination of the placenta is often omitted because of lacking resources, training, and protection against contaminated blood, and poor understanding of its clinical importance in identifying the cause of death. Cultural practices may also prevent such examinations, as e.g. in China, Pacific Islands and West Africa, where the placenta may be of high symbolic and spiritual importance, and should be eaten or buried at specific sites.

Although this report focuses on the specific needs to capture data on stillbirths, neonatal conditions and deaths should be included in the same system to ensure a full picture of perinatal mortality, linked to maternal conditions and causes of death. In particular, causes and mechanisms of neonatal deaths within 12-24 hours of life are generally the same as for intrapartum stillbirths [81].

### Classification of causes of death and associated conditions

Classification systems for stillbirths may be useful if designed for comparison of communities, for audit of care, and for planning and monitoring prevention and research. Besides the ICD system, there is no international consensus on a classification system for stillbirths. The ICD aims to classify the underlying cause of death, and when not available, the condition most likely to have resulted in death. It is used extensively in low-income countries, for example, to report perinatal mortality to WHO. The ICD system was developed to allow the systematic coding, analysis, interpretation, and comparison of mortality and morbidity, but it is not adapted specifically to stillbirth. Thus, the current ICD-10 does not fully recognize the stillborn infant (with cord, placenta, and membranes) as an individual entity with its own diseases, conditions, and events to be registered separately from the mother. There are limited codes for conditions specific to the perinatal period in the O codes ("pregnancy, childbirth, and puerperium," from the maternal perspective) and P codes ("certain conditions originating in the perinatal period," from the fetal perspective), where adequate coding for stillbirths should be found. The placental codes in particular are incomplete and can only be used for a few of the placental pathologies affecting pregnancy outcomes. This lack of coding options is further complicated by our incomplete understanding and continued controversy regarding various placental findings and their causality in stillbirths. There is also considerable overlap between O and P codes that may result in confusion for allocation. WHO advocates the accurate use of ICD codes to monitor the quality of registrations of causes of death, but the range of incomplete categories of stillbirths in ICD-10 suggest that for now, other indicators of quality must be defined. The current revision of the ICD system (ICD-11) is expected to be released in 2013.

With these limitations in the ICD system, and with the many different purposes of classification, a number of alternative and mostly mutually incompatible classifications for stillbirths have been designed over the past decades [82]. A classification system should be an information management tool [49,50]. Depending on the purpose (local health care monitoring, planning, or research), different classification systems may be helpful, and a perfect all-purpose system is unthinkable. Although classifications are typically used in low-income countries to identify the main areas of preventive efforts [83,84], none of them has been rigorously tested to determine the effects on stillbirth prevention and rates after implementation. A core classification system for the main categories of causes of death and associated risk factors and conditions in stillbirths, as collected in our proposed dataset template, is nonetheless the starting point. For international comparisons to be meaningful, the most prevalent low-income country-related causes and conditions must be included in the classifications. Most current classifications have been created for high-income countries and implemented in low- and middle-income countries without modifications. Typically, a system for a high-resource setting will not be poised to adequately manage information on, for example, intrapartum deaths or infections such as malaria and syphilis. A base system needs some fundamental elements, and the corresponding characteristics of the few that have been tested in low- and middle-income countries are listed in Table 1.

**Table 1:** Classification systems and their characteristics for use in low- and middle-income countries

Systems tested in low- or middle-income countries	Number of categories of stillbirths^a^	Are intrapartum events captured in subcategories?	Does the system aim to capture underlying cause?	Are the main categories consistent with underlying cause?	Resources desirable for use^b^	Does the system separate unknown from unexplained?	Agreement tested (Kappa score or level of agreement)	References
Aberdeen	8-0-0	no	yes	no	B	no	0.35-0.97	[49,84,93-95]
CODAC	10-94-577	yes	yes	yes	B, C	yes	0.65-0.94	[49,50]
CODAC Simplified	10-30	yes	yes	yes	B, C	yes	no	[50]
ICD-10	17-134	yes	yes	yes/no	B	no	no	[23]
Nordic Baltic	13-0-0	no	no	no	A	no	0.85	[84]
Pattinson^c^	12-48-0	yes	no	no	B	no	no	[96,97]
PSANZ-PDC	11-52-33	yes/no	yes	yes/no	B, C	yes	0.63-0.90	[49,86]
ReCoDe	9-28-1	yes/no	no	no	B, C	yes	0.51	[49,98]
Tulip	6-24-7	no/yes	yes	yes	B, C	yes	0.74-0.86	[49,87]
Whitfield^c^	12-15-2	no	yes	no	B	no	no	[95,99]
Wigglesworth	5-0-0	no	no	no	A	no	0.25-0.85	[49,84]

^a^Number of main categories followed by subcategories.

^b^A = little investigations and no placental examinations necessary; B = some clinical and pathological investigations necessary or desirable; C = placental investigations necessary or desirable (placental conditions included in the classification).

^c^Modified versions of the Aberdeen classification.

Acronyms: CODAC: Causes of Death and Associated Conditions, PSANZ-PDC: Perinatal Society of Australia and New Zealand Perinatal Death Classification, ReCoDe: Relevant Conditions of Death.

**1) Compatibility with ICD**. The aim should be to supplement the ICD system, and to ensure compatibility, a tracking system for stillbirths should adhere to the concepts of capturing the underlying causes. Although additional information, such as the specific identification of potentially preventable associated conditions, may be beneficial, its inclusion should not make the system incompatible with ICD concepts.

**Figure 5:**
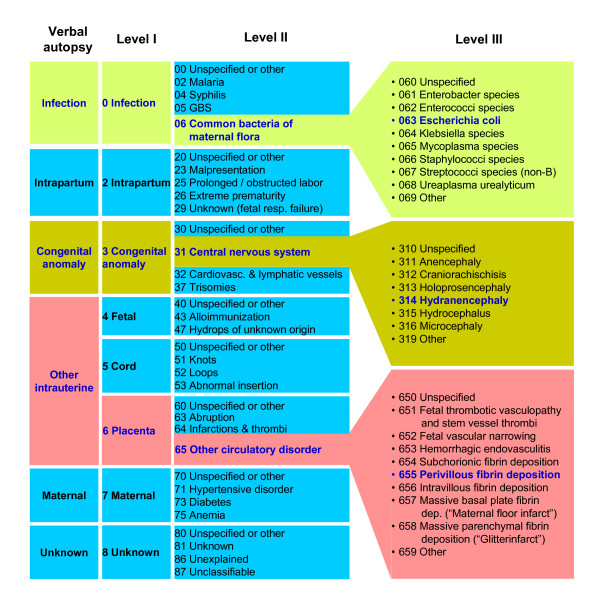
**Causes of death in stillbirth**. Expandable layers of causes of death according to the resources available for evaluations of stillbirths. Select categories of the CODAC classification system [50] used for illustration.

**2) Expandability of classifications**. To be perceived as useful in all settings, the basic categories should allow the simplicity of data collection from verbal autopsies. Yet, these categories should also be expandable to the individual diagnoses registered in high-resource, high-expertise settings to promote consistency across populations and facilitate knowledge transfer. The Causes of Death and Associated Conditions (CODAC) classification is used as one such example for illustration in Figure 5, where the first case could be identified by verbal autopsy as an intrauterine infection with foul-smelling amniotic fluid and maternal fever, and diagnosed in other settings as an ascending *E. coli *infection confirmed by cultures from internal fetal tissues. Second, an obvious congenital anomaly of the head could be subclassified as hydranencephaly in a specialized center for perinatal pathology. Third, a case identified in low-resource settings as a small baby with a rubber-hard placenta with white-gray lesions, can be identified by perinatal pathologists as massive perivillous fibrin depositions. Using expandable classifications, data collection is possible for all, yet none are restricted by a too-simplistic system from preserving more complex information.

**3) Capture of intrapartum events**. A data system should include categories to distinguish items on antepartum and intrapartum conditions that are targets of preventive efforts in antenatal and intrapartum care, as in the dataset we present here. Specifically, it should not only state that death occurred in the intrapartum period, but also capture the actual intrapartum events. As an example, the Wigglesworth system mainly focuses on timing of death, which is favoured in many low-resource settings because its simplicity allows any birth attendants to use it irrespective of his or her educational level. Yet, in its original version, it did not handle any information other than timing and congenital malformations, and the simplicity of the system does not outweigh the loss of information [84,85]. A classification system without information on intrapartum events will be of little use in one third of stillbirths globally.

**4) Capture of placental conditions**. The conditions affecting the stillborn infant that are related to the adjoining cord, placenta, and membranes need to be recorded. Of particular importance is managing the information on the placenta, as this is lacking in the current ICD.

**Figure 6:**
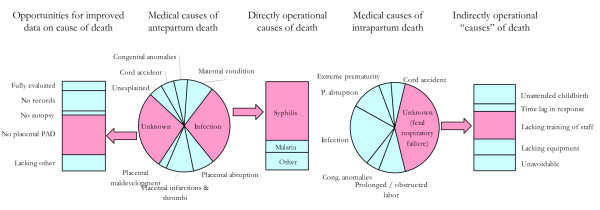
**Operational causes of death**. Examples of causes of antepartum and intrapartum deaths that may be targeted directly for stillbirth prevention (e.g., syphilis) or indirectly to improve clinical quality of care or ability to capture data for further improvement. PAD: pathological-anatomical diagnosis.

**5) Ability to differentiate unknown and unexplained events**. While infections like syphilis provide direct causes of death as targets for prevention and should be included, the category of "unknown" events in antepartum and intrapartum deaths also provides indirect operational causes with opportunity for improvement that should be targeted, as shown in Figure 6. The classification system should enable differentiation between unknown and unexplained causes of death to allow meaningful analysis that takes into account the proportion of items that are unknown in the dataset. Ideally, one would expect unexplained stillbirths to be stillbirths "(...) unexpected by history and in which a thorough autopsy (...), together with gross and histological examination of the umbilical cord, placenta, and membranes, fails to demonstrate an adequate cause of death." [67].

Of the existing classification systems that have been tested in low- or middle-income country settings, only the current versions of the Perinatal Society of Australia and New Zealand Perinatal Death Classification (PSANZ-PDC) [86] and Causes of Death and Associated Conditions CODAC [50] comply with all five of these requirements, although the basic level of the main categories may have to be simplified further to be used with verbal autopsy data only. The summary in the Table 1 would indicate that Tulip [87] requires only the addition of an explicit category for intrapartum events to comply (intrapartum causes are embedded in other categories in Tulip). Tulip is uniquely organized to capture information from a high-resource, high-expertise setting with focus on the detailed diagnosis of malformations and placental abnormalities [64,87], and it is not intended for use in low- and middle-income country settings.

The two systems that seem to be the best options to supplement ICD, CODAC and PSANZ-PDC, are among the systems in which some clinical, pathological, and placental investigations would be necessary or desirable to ensure consistency in coding (Table 1). This follows from the inclusion of the placenta as an entity to capture. To set lower standards and accept a lack of data on conditions contributing to two thirds of antepartum stillbirths in high-income countries [19,49,64] is not a viable alternative. Use of the more elaborate systems may introduce misclassification in a setting with low resources. For instance, placental and infectious causes may be under-diagnosed, classified instead as unknown causes. However, although the value of placental histopathology and autopsy is undisputed in high-resource countries [88-90], the potential for prevention (for society and for the mother's subsequent pregnancies) associated with elaborate post-mortem testing in low-income countries is unclear, and the minimum level of investigation needed to classify a cause of death as unexplained rather than unknown in low- and middle-income country settings must be defined. For the purpose of data collection from verbal autopsies, categories such as "fetal," "cord," and "placenta," typically found in many classifications, could easily be combined, e.g., into "other intrauterine," as exemplified in Figure 5. The underlying cause of fetal hydrops or location of an intrauterine infection as either fetal, cord, or placenta will be particularly difficult to decide.

The number of categories per system reflects the amount of information captured by that system (Table 1), but it does not necessarily reflect the applicability of these categories for analyzing stillbirth scenarios in all settings. The ease of use with little information versus more information requiring more resources for investigations must be balanced by the local knowledge of its intended use. Interestingly, testing the ease of use and ability to capture pertinent information on stillbirths in both high- and low-resource settings demonstrated that the complexity of the three systems mentioned previously was not associated with reduced user-friendliness. On the contrary, health professionals coding stillbirths seem to prefer having the opportunity to code exact information rather than registering information in broader and more vague categories [49,50], and all complex systems also have broader categories for comparisons that are detailed in subcategories.

It is time that traditional classifications such as Wigglesworth be celebrated for past achievements and substituted with classification systems that manage the information currently available. Several dozen classification systems exist already. However, some of these systems still require testing, and new systems must be developed specifically to handle verbal autopsy data. Such improvements in data management are essential to informing targeted interventions to prevent stillbirths. As the ultimate goal is stillbirth prevention, future research in the development and use of classification systems should aim to document its usefulness in this respect.

## Summary

Scaled-up of coverage of registration of births is needed. Vital registrations and health information systems and other large Demographic and Health Surveys should be adjusted to accommodate registration of stillbirths. Tools for verbal autopsies must be revised to improve capture of causes and events related to stillbirths. The WHO definitions of stillbirth for reporting should be implemented globally, and registrations should capture stillbirths of lower weight and gestation than the intended reporting limits. Pregnancies should be registered before the perinatal period to help overcome the barriers in stillbirth registration and enable prevention efforts in antenatal care. Causes of death in stillbirths must be monitored, and better detection of the most prevalent causes--in particular, infections and placental pathologies--is needed. The ICD system's O and P codes need to be adjusted and supplemented to capture the causes identified. A universal approach to stillbirth classification is optimal and warrants focused attention.

The *BMC Pregnancy and Childbirth *issue on stillbirth prevention illustrates how specific causes of stillbirth can be prevented [3,6,41,91,92]. None of this will happen without data. Development and evaluation of prevention efforts require registration of all stillbirths, the characteristics of the pregnancies affected, the timing of death, the underlying causes, and the quality and availability of care for each individual. Stillbirths need to count. With careful collection and analysis of these data, the numbers will speak for themselves.

## Abbreviations

WHO: World Health Organization; ICD: International Classification of Diseases.

## Competing interests

The authors declare that they have no competing interests.

## Appendices

Appendix 1 - Success criteria for collection and reporting of stillbirth data

• Consistent use of definitions between institutions

• Systematic approach to capture pregnancies and out-of-hospital stillbirths

• Capacity and training in examinations and testing procedures to identify cause of death

• Easily accessible electronic files for registration

• Training in stillbirth registration and classification

• Dedicated and motivated staff for registrations

• Feedback and other incentives to motivate staff

• Local adaptations of a sustainable system for registering and reporting

• Quality indicators developed for local needs

• Regional or national data collection and analysis

Appendix 2 - Egypt: Assiut University Hospital

The hospital center in Assiut is a tertiary university hospital that receives women from low to middle socioeconomic levels. In 2007, the hospital attended 13,398 deliveries and 6550 antenatal care clients. There is 50%-75% coverage of the care system in the area.

Pregnancies are captured from 22 weeks gestation. The current system can also register stillbirths from 28 weeks gestation (or 1000 g or 35 cm) including intrapartum deaths. Using this definition, the hospital stillbirth rate is 70/1000.

No specific person is responsible for reporting stillbirths in particular. However, systematic reporting on stillbirths is included in the general statistics of the delivery unit, which provides the stillbirth rate. The outcome of pregnancy is reported in the medical file by the attendant obstetrician, but these files are not easily accessed for registrations. Therefore, the hospital is moving towards introducing the recording of labor and delivery data into a computerized database. This move would facilitate data capture and strengthen the quality of data in general and in particular, the quality of reported pregnancies and stillbirths. The main challenge of such a system is sustainability within available resources. Data entry is performed by residents who need to be motivated and to see the usefulness of the work in order to achieve quality in the reporting.

The proposed system of registration could be implemented at the Assiut Hospital. An anticipated challenge would be the necessity to train data collectors in ICD-10. Potential feasible additions to the proposed dataset for monitoring includes collection and laboratory analysis of cord blood to identify blood group incompatibilities, genetic analysis, and photographs to document the findings of macroscopic exams.

Appendix 3 - Thailand: Srinagarind Hospital

Srinagarind is a 1000-bed, urban teaching hospital located in Khon Kaen in the Khon Kaen province of Thailand. The hospital provides comprehensive obstetric care. Each year, 2500-3000 women give birth in Srinagarind, and it provides antenatal care to 2500 women. The antenatal care clinic opens weekdays from 9:00 to noon, and the high-risk pregnancy clinic opens from 13:00 to 16:00.

The hospital's strategy is to capture pregnancies from <22 weeks and stillbirths based on the 22-week definition (500 g or 25 cm). With this definition, the stillbirth rate is 7/1000. The stillbirth capturing system identifies intrapartum deaths, which represent 3/1000 births.

Registered nurses register all births (live and stillbirth), and one senior registered nurse is responsible for reporting. There is no lower limit on registration and reporting of live births. Residents are responsible for reporting the monthly statistics in the departmental meeting. Information on all births, including complications, is entered into computer files that can be readily assessed.

The proposed system of registration could be implemented at Srinagarind Hospital, Khon Kaen University. All items are applicable in this setting would be integrated with neonatal death registrations. Additions to the dataset might include the ability to distinguish between vacuum and forceps in assisted vaginal deliveries and separate categories for maternal anemia and pre-eclampsia, preterm labor, and fetal distress.

Appendix 4 - South Africa: East London Hospital Complex

Located in East London, this urban hospital complex is a second-line birthing institution equipped with 1676 beds, of which 60 are gynecology beds and 180 obstetric beds. It receives mainly women from low socioeconomic groups who cannot afford private care. The number of births per year is 13,000-14,000, and the hospital provides antenatal care for the same population. It has high coverage of both antenatal (>80%) and intrapartum (>90%) care. The hospital uses the stillbirth definition ≥ 500 g (weight only, no gestational age or length) for registration, and the stillbirth rate ≥ 1000 g is 23/1000. The intrapartum stillbirth rate is approximately 7/1000.

A significant strength of the hospital's registration program is its participation in the well-structured national Perinatal Problem Identification Program (PPIP, http://www.ppip.co.za). Doctors or midwives fill in the individual perinatal death report, while a dedicated statistics nurse registers births and checks that all births recorded in the labor ward are accounted for. Data are submitted monthly to a national PPIP database. PPIP differs from the proposed classification approach in that it does not collect data specifically on intrapartum vs. antepartum deaths, but on four categories: "stillborn, alive on admission," "fresh stillborn, dead on admission," "stillborn, admission status unknown," and "macerated stillborn." The sum of the three first categories is reported as an approximation of intrapartum death rates.

A weakness of the hospital's current system is the inability to capture deaths outside the hospital in the communities served and, thus, a lack of information on perinatal deaths occurring at home. A second weakness is the use of a manual counting system and not an electronic maternity database. The latter would be a feasible and major opportunity to improve data collection.

In this setting, there are few obstacles to capturing data on stillbirths systematically, and extending registrations to a maternity database remains a question of prioritizing resources in a setting where skilled staff shortage is the main limitation of the health care system. A maternity database would not directly lead to improved capture of stillbirths in home deliveries. Some could be captured with access to death registration data, but some are not reported at all by the families.

A specific disincentive to the registration of stillbirths in South Africa is that registered stillborn babies require a formal burial, which places a large financial burden on the family. Even in the case of in-hospital stillbirths of borderline weight, hospital staff may assist the family to avoid the cost of burial by recording the death as a miscarriage.

## Authors' contributions

JFF drafted the manuscript in consultation with SJG. SJG reviewed existing classification systems in the table. AB and MM collected and drafted the experiences on data collection in Appendices 1, 2, 3 and 4. All authors edited drafts of the manuscript and read and approved the final manuscript.

## Pre-publication history

The pre-publication history for this paper can be accessed here:

http://www.biomedcentral.com/1471-2393/9/58/prepub
